# Examining income-related inequality in health literacy and health-information seeking among urban population in China

**DOI:** 10.1186/s12889-019-6538-2

**Published:** 2019-02-21

**Authors:** Chengxiang Tang, Xueji Wu, Xiongfei Chen, Bingying Pan, Xiaocong Yang

**Affiliations:** 1 School of Public Administration, Guangzhou University, Guangzhou, Guangdong China; 2 Department of Primary Public Health, Guangzhou Center for Disease Control and Prevention, Guangzhou, Guangdong China

**Keywords:** Inequality, Health Literacy, Health Information Seeking, China

## Abstract

**Background:**

Health literacy and health-information seeking behaviour (HISB) play vital roles in health outcome improvements. This study examines the extent of income-related inequality in health literacy and health-information seeking as well as the contributions of the main socioeconomic determinants in China.

**Methods:**

We analysed representative data of participants aged over 18 years as well as older adults from the Guangzhou Community Health Survey. A concentration index (CI) was used to quantify the degree of income-related inequity in health literacy and health-information seeking. Probit regression models were employed to decompose the CI into the contributions to each factor.

**Results:**

Results showed a significant pro-rich distribution of adequate health literacy (CI: 0.0602, *P* < 0.001; horizontal index [HI]: 0.0562, *P* < 0.001) and HISB from healthcare professionals (CI: 0.105, *P* < 0.001; HI: 0.0965, *P* < 0.001). The pro-rich distribution of health literacy was mainly attributable to education background (contribution: 54.76%), whereas income inequalities contributed most to the pro-rich distribution of health-information seeking among an urban population (contribution: 62.53%).

**Conclusion:**

Public interventions in China to reduce inequality in health literacy and HISBs among the urban population, coupled with easily accessible information sources on health, warrant further attention from policymakers.

## Background

With a growing ageing population and prevalent transition to chronic non-communicable disease, patients worldwide are increasingly encouraged and often expected to be involved in healthcare interaction with physicians [1, 2]. Specifically, this requires more than just a physician verbally giving a patient the diagnosis and medication and includes interactive communication to empower patients to initiatively ask questions and seek information, thus informing shared decisions regarding health and healthcare. The evolutional patient–physician relationship enables shared decision making between them, so that health outcomes of patients can be improved through the development of health literacy and reduction of barriers to health-information seeking [3, 4]. In brief, equitable health literacy and adequate health-information seeking comprise a key area in today’s health system.

Individuals with limited health literacy may still be misguided by incorrect source of health information and struggle to navigate health-information-seeking behaviours (HISBs) [5]. Low health literacy has been associated with poorer health outcomes and poorer use of healthcare services [6–8]. Moreover, studies observed an array of socioeconomic disparity related to low health literacy and improper health-information seeking [9]. In an attempt to ensure equitable access to healthcare services, many policymakers striving to improve health systems have shared a goal of reducing health disparities by focusing on health literacy for those who have the same needs for healthcare, regardless of their socioeconomic background [10–12].

In developed countries, the overall evidence on the relationship between health literacy, health-information access, and disparities is still mixed and limited [13, 14]. Studies have varied with regard to the precise nature of assessing this relationship; as a result, a specific description of the nature of the disparity and potential explanatory pathways on how income and education contribute to such disparities require further investigation [13, 15]. Despite China reforming its healthcare system to build an integrated and cooperative primary health system [16], no evidence exists that the current situation gives a promise on equity of health literacy and health-information seeking among the population, even in urban China. Equity in health can only be attained if individuals are equipped with nearly the same level of health literacy and have equal access to healthcare services [17]. Therefore, it is vital to assess income-related equity in health literacy and HISBs, because such equity represents an opportunity to receive equal healthcare and a potential opportunity to improve health outcomes.

Health literacy mainly influences health outcomes in three ways. First, the navigation skills and perceived barriers will impact the access to healthcare and the utilisation. Second, the health-related knowledge or beliefs will affect medical decision and discussion in interactions between patients and physicians. Third, self-treatment and prevention behaviours are influenced by motivation, solutions to health problems, and health-related skills [18]. The oft-cited definition of health literacy generally consists of medical and public health scopes. The former one typically focuses on conceptual knowledge and interaction skills between doctors and patients, whereas public health literacy is linked to health education and improvements of public health interventions for populations. However, health literacy has been extended into more dimensions at present, for example, functional health literacy often refers to knowledge of risks, health services, and compliance with prescribed actions; interactive health literacy is used to indicate an improved capacity to act independently, and improved motivation and high self-confidence; while critical health literacy may related to improve individual resilience to social and economic adversity. Therefore, health literacy has been considered as an important process or a pathway that makes people improve health-related knowledge and skills, and thus generate better health behaviours in lives. HISB refers to the ways in which individuals seek information regarding their health, such as risks, illnesses, and health-protective behaviours to improve their health [19]. The associated factors of HISB include the following: (1) health-information features, such as the concepts, contents, credibility, or accuracy; (2) personal characteristics, such as gender, age, education level, income, beliefs or health status; and (3) external environment (i.e., whether it is supportive or not), such as family or workplace supports, social security system, and richness of information carriers. Many people’s lifestyles can been improved by actively seeking health information [20]. That is, people who more actively seek medical information are more likely to visit doctors and undergo more medical examinations [21]. However, people do not always behave actively regarding doctor visits. For example, with the rapid development of the internet, in Mainland China, more people tend to seek health information online because it is easier and more accessible than seeing a doctor. Inaccurate health information sources and inability to find and understand that information are the main barrier to obtaining professional information and subsequent healthcare seeking. This leads people to not trust doctors and subsequently not to visit them. Therefore, people’s behaviours are influenced by the interaction among the aforementioned factors related to HISB.

In this study, we examined the inequality and inequity in health literacy and information seeking from healthcare professionals in China, using representative, cross-sectional household survey data. This study also examined factors associated with health literacy and health information seeking among an urban population of Chinese adults.

## Methods

### Study data

The data in this study were derived from a repeated cross-sectional survey entitled Guangzhou Community Health Survey (GCHS 2013). This survey used a stratified multistage cluster sampling design to recruit participants between May and June 2013. First, a certain number of community health services centres or township hospitals (subdistrict or township level) were randomly selected from each district (county level); second, at least five communities or villages within the subdistrict areas were randomly selected; third, the survey accessed numerous households from a list of household names. All family members who had lived in Guangzhou for at least 6 months were eligible for interview. The survey was designed to represent the permanent residential population in Guangzhou area and was used in published studies [22, 23]. A total of 71 communities or villages and 6677 households were included in GCHS 2013. The final dataset included 21,860 individuals who were over 18 years old and excluded pregnant women.

We generally conducted the survey in three steps. First, we posted advertisements to spread information of this survey in these communities 30-days ahead of the interviews. Second, we obtained census data from local official organisations to extract sample individuals who were registered in the census data and had lived in Guangzhou for more than 6 months. Third, the survey was a face-to-face interview in which, all interviewers are trained by physicians based on standardised protocol before the survey. Sampled households who gave written inform consent and verbally agreed to join the survey were scheduled to be interviewed by telephone in advance. Ethical approval was obtained from the Guangzhou Centre of Disease Control. This survey was specially funded by Health Bureau of Guangzhou [23].

The questionnaire was designed by the National Health Department, but some questions were modified for appropriate localisation. The final version of the GCHS questionnaire comprised three parts: (I) Individual level data, including individual health condition (e.g., self-reported diseases, including diagnosis of hypertension and type 2 diabetes (TB2) control and treatment); health risk factors and knowledge of health and health behaviours, awareness of hypertension and other non-communicable diseases, underlying risk factors of physical inactivity, tobacco use and unhealthy alcohol use; and a physical test, including height, weight, waistline, hipline, systolic blood pressure, and diastolic blood pressure; (II) Family and socioeconomic background, including number of family members, house type, annual income, medical expenses, and health and education resources; and (III) Community level data, including geographic features, population and birth rate [23].

### Variables

We followed a publication to construct a dependent variable that was used to measure individuals’ health literacy because our survey as a long questionnaire, but include no comprehensive measurement of health literacy [24–26]. Following the operational definition of knowledge–attitude–behaviour (KAB) theory extensively used in health education and our review of other instruments, we selected some commonly used indicators and adapted them to the lower educational level of the surveyed participants in China [27–29].^1^ Table 1 shows these indicators in relation to four dimensions of health literacy. First, health knowledge dimension involve knowledge that people oft-cited in daily life such as risk factors, prevention and treatment strategies for health conditions, and the effect of chronic problems. Second, health-information presentation skill involves the complexity of reading comprehension and numeracy in medical settings, as measured by answering questions regarding contraindication, blood pressure identification, and body mass index range. Third, we used indicators such as views on TB2, smoking, and regular physical examination to measure health belief. Fourth, Health behaviour may refer to regular and patterned behaviours related to health, including health information seeking and health management [26].

**Table 1:** Health literacy dimensions and related indicators

Dimensions	Indicators and questions
Health knowledge	Is a high-salt diet associated with hypertension? (Yes/No)
	Is obesity associated with hypertension? (Yes/No)
	Which one do you consider a more balanced diet? (1) Diet with variety and balanced mix of nutrients, (2) Diet with the same amount for every kind of nutrient, (3) Diet with high nutrition, (9) Unknown
	Is mental stress related to hypertension? (Yes/No)
Health-information presentation skill	Have you ever joined a health workshop organised by a community facility? (Yes/No)
	What is the range of an adult’s body mass index? (1) 18.5-23.9, (2) 24-27.9, (9) Unknown
	Do you know the diagnostic criteria for hypertension? (1) below 90/60 mmHg, (2) above 140/90 mmHg, (3) above 160/96 mmHg, (9) Unknown
	Patients with Type 2 diabetes can be healed by one course of treatment, so they do not need to take long-term medications. (True/False)
Health belief	Smoking causes cardiovascular disease. (True/False)
	Patients with Type 2 diabetes mainly rely on medications. (True/False)
	Do you pay attention to your intake of cooking oil (fat)? (Yes/No)
	Healthy people do not need to receive regular physical examinations. (Yes/No)
Health behaviour	Do you take the initiatives to access health knowledge? (Yes/No)
	Do you have any physical activities in leisure time (for example, walking, dancing, gardening, hiking, swimming) at a moderate or vigorous intensity for at least 10 minutes at a time? (Yes/No)
	What actions do you usually take when you are ill or are feeling uncomfortable? (1) Self-medication, (2) Visit a doctor for consultation, (3) Self-treatment and look for medical advice from a doctor

To construct a binary variable to measure health literacy, we then calculated a single factor by running an exploratory factor analysis on the four means of the four dimensions of HL. A single factor with an eigenvalue greater than one was identified, accounting for 71% of the variance in the correlation matrix. The HL factor score was calculated using a regression equation, which was assumed to accurately reflect the level of HL with an inclusion of the weighting factors against dimensions. Internal consistency reliability for the four items was determined by computing Cronbach’s alpha (= 0.771). Following the Chinese Adult Health Literacy Questionnaire, the single HL was classified as high and low that is a binary variable for high HL (= 1 if factor score >= median) and low HL (= 0 if factor score < median), in terms of the criterion of the median score for a revised HL scale based on the Test of Functional Health Literacy in Adults [26, 27, 30]. In addition, the other dependent variable HISB is measured by the question “Which of these are considered to be your primary source of health-information seeking?” Despite the absence of a consistent definition of HISB, most authors described it as an influencing factor or a component of health behaviour [19].

### Statistics analysis

To measure inequality and explain socioeconomic-related inequality in the health literacy and HISBs, we employed a conventional method — calculation of concentration index (CI) and drawing a concentration curve [10, 31]. In a concentration curve of HISBs, the cumulative percentage of respondents with adequate HL or respondents who considered healthcare professionals as their primary source of health-information seeking (as the y-axis) was plotted against the cumulative percentage of the population ranked by per capita annual income (economic status) from the lowest to highest (as the x-axis). The CI was defined as two times the area between the concentration curve and the line of equality (the 45° line). A concentration curve lying below the line of equality pointed out that health-related status or behaviour was concentrated among the richer class [32]. We also obtained 95% confidence intervals for the CI and the associated P-values in analysis. It is clear to interpret that when the CI was significantly smaller (larger) than 0, poor (wealthier) individuals were more likely to possess adequate HL or seek health information from professionals. Generally, the CI adopted a negative value when the curve rose above the line of equality, and a positive value when it fell below.

To further measure the inequity in health literacy and information seeking, we estimated the horizontal index (HI). CI measured the extent of socioeconomic-related inequality in two dependent variables. However, because of differences in the need for health literacy and information seeking across different socioeconomic classes, inequality does not always reflect inequity. Therefore, HI reflects socioeconomic differences in health literacy and information seeking while controlling for the effects of biological needs. HI was calculated by subtracting the absolute contributions made by need factors from the CI. The need could be represented by gender, age, self-assessed health status, or chronic diseases (measuring and testing).

We conducted a decomposition to assess the extent to which individual factors contributed to income-related health inequality. A positive (negative) contribution indicated that the specific independent variable operated towards pro-rich (pro-poor) distribution of health literacy or health-information seeking. The Probit model was used to decompose the CI, in which the contribution percentages of each factor to the CI were calculated [10]. We adopted a similar framework in decomposing contributions to healthcare utilisation, in which independent variables were categorised into need factors, non-need factors, and economic status. First, need factors or biological determinants are used as a proxy for health literacy and health-information needs, which include age, gender, and morbidity [32]. The morbidity variable in this study comprises eight commonly reported diseases and symptoms. Second, non-need factors include variables regarding socioeconomic background for HL and HISB, which include the education level of the individual (primary or lower, secondary or college level) and marital status (never married, currently married, divorced/separated/widowed). Third, economic status was described by household annual income per capita, which was converted into the natural logarithm form [33]. Statistical analyses were performed using Stata software package of version 14.1.

## Results

Table 2 presents the characteristics of the individuals (*n* = 17,290) included in our analysis. In total, over half of the individuals (55.5%) were rated as having adequate health literacy. A quarter of the sampled individuals considered healthcare professionals (26.2 %) as their primary source of health information. More females than males completed the survey (54.6% females; 45.4% males). More respondents reported a secondary level of education (53.2%) and married status (78.8%) than they did higher education (tertiary level: 21.7%) and single status (never married or other status: 21.2%). The average household income per capita (27,616 RMB) in our sample was nearly equal to the income reported in the official statistics (26,955 RMB) [34]. Therefore, this sample is representative of residents in Guangzhou city.

**Table 2:** Characteristics of the Study Sample from Guangzhou (2013 data)

		N (=17290)	%
Dependent variables			
Adequate health literacy		9599	55.52
Health information seeking from health professionals		4530	26.20
Need factors			
Age	Under 30 years	2749	15.90
	30-59 years	9778	56.55
	60 and above	4763	27.55
Gender	Male	7837	45.33
	Female	9453	54.67
Self-reported diseases/symptoms	Hypertension	2858	16.53
	Diabetes	1020	5.90
	Heart	370	2.14
	Gastritis	396	2.29
	Arthritis	357	2.06
	Hyperlipidemia	551	3.19
	Pulmonary	72	0.42
	Sleepless	7538	43.60
Non-need factors			
Education level	Primary or lower	4332	25.05
	Secondary level	9203	53.23
	College level	3755	21.72
Marital status	Never-married	2243	12.97
	Currently married	13628	78.82
	Divorced/separated/widowed	1419	8.21
Household economic status			
Household income per capita in RMB, mean (SD)		27615.91	(39625)

**Fig. 1:**
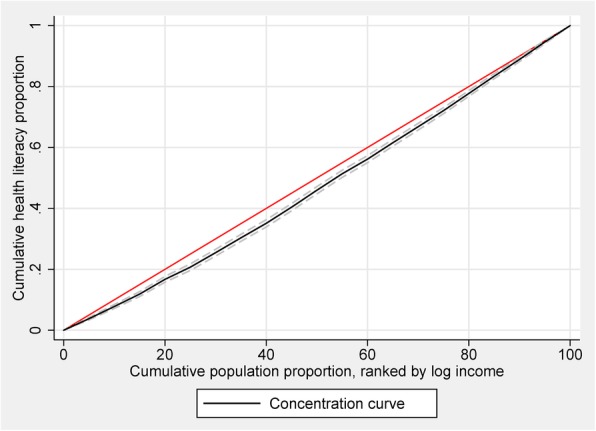
Concentration curve for HL

**Fig. 2:**
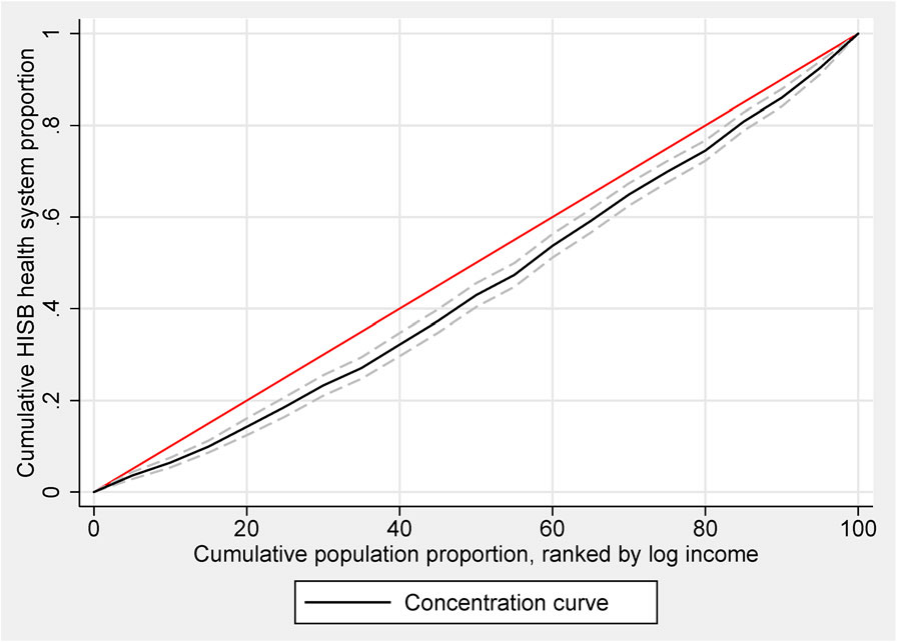
Concentration curve for HISB-HC

**Fig. 3:**
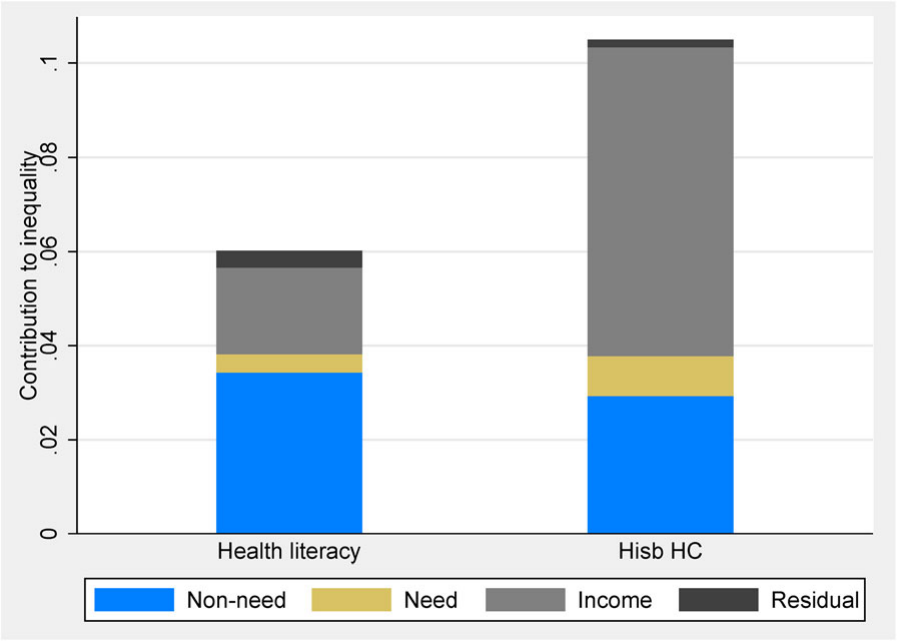
Contributions of need, non-need, and income factors to CI for HL and HISB

Figure 1 plots the concentration curves for probability of being an individual with adequate health literacy. Figure 2 plots the concentration curves for probability of being an individual who mainly relies on healthcare professionals as a source of health information. The results indicated a significant pro-rich distribution of adequate health literacy (CI: 0.0602, *P* < 0.001) and HISBs from healthcare professionals (CI: 0.105, *P* < 0.001). Figure 3 depicts the inequality in health literacy and information seeking decomposed by need, non-need, household income factors, and residual term. Non-need factors and household economic status accounted for the pro-rich concentration of health literacy and health-information seeking. Compared with the left bar of decomposing health literacy, income indicator accounted for the largest part of health-information seeking from healthcare professionals.

Table 3 presents the detailed contribution of need, non-need, and household income factors to inequality in health literacy and health-information seeking. The pro-rich distribution of health literacy was mainly attributable to education background (contribution: 54.76%), whereas income inequalities contributed most to the pro-rich distribution of health-information seeking among the urban population (contribution: 62.53%). After deducting the inequality induced by need factors, horizontal inequity was significantly pro-rich in adequate health literacy (HI: 0.0562, *P* < 0.001) and HISBs from healthcare professionals (HI: 0.0965, *P* < 0.001).

**Table 3:** Contributions to inequality in health literacy and information seeking from the health system

	Health literacy			Health information seeking from health system		
	Concentration index	Contribution	Percentage contribution	Concentration index	Contribution	Percentage contribution
CI for adequate health literacy		0.0602			0.105	
Horizontal Inequity (HI) index		0.0562			0.0965	
Residual		0.0035			0.0016	
Need factors						
Age						
Under 30 years	Ref.					
30-59 years	0.0452	0.001	1.7344	0.0456	0.003	2.8194
60 and above	0.0406	0.0003	0.5711	0.042	0.0012	1.1009
Gender						
Male	Ref.					
Female	0.0034	0.0002	0.3267	0.0027	0.0002	0.2232
Self-reported disease						
Hypertension	0.0498	0.0006	0.9983	0.0493	0.0013	1.2076
Diabetes	0.1018	0.001	1.6944	0.0987	0.0023	2.2121
Heart	0.1009	0.0001	0.2244	0.1014	0.0002	0.2003
Gastritis	-0.1254	0.0004	0.6179	-0.1271	0.0007	0.6699
Arthritis	0.1954	0	0.0536	0.2054	-0.0001	-0.1394
Hyperlipidemia	0.0752	0.0003	0.4157	0.0707	0	0.0371
Pulmonary	-0.0758	-0.0001	-0.1031	-0.0829	-0.0001	-0.1222
Sleepless	-0.005	0	0.0314	-0.0046	-0.0001	-0.1077
Non-need factors						
Educational level						
Primary or lower	Ref.					
Secondary level	-0.0856	-0.0181	-30.156	-0.0864	-0.0075	-7.1422
College level	0.2342	0.0511	84.9174	0.2341	0.0332	31.6699
Marital status						
Never-married	Ref.					
Currently married	-0.0512	0.0006	0.9794	-0.0518	0.005	4.752
Divorced/separated/widowed	0.0223	0.0007	1.1733	0.0224	-0.0015	-1.3981
Household annual income (log of income per capita)	0.0439	0.0184	30.6448	0.0438	0.0656	62.5293

Decomposition based on Probit model results. Sample weights applied. Statistically significant estimates are in bold type (*P* < 0.05)

## Discussion

The objectives of this study were to investigate the extent of income-related inequality in health literacy and health-information seeking, and the contributions of the main socioeconomic determinants in China. Our three main findings were as follows: (1) a significant pro-rich distribution of adequate health literacy and HISBs from healthcare professionals; (2) the pro-rich distribution of health literacy was mainly attributable to education background; and (3) income inequalities contributed most to the pro-rich distribution of health-information seeking among the urban population.

Numerous studies have documented inequalities in health-related fields. For example, Zou Nan found that income inequality did not have a significant impact on individuals' risks of having health problems [35]. Another study looked at the income-related health inequality between the rural and urban population by using self-assessed health and physical activity limitation [36]. Other research has focused on income-related inequality of healthcare utilisation and access to healthcare in China [37, 38]. However, the relationship of health literacy and HISBs with income distribution is not well understood in China. This paper sought to address the gap in the literature regarding health inequality.

The findings revealed a significant socioeconomic disparity in health literacy. Compared with the high-income population, the low-income population had more difficulty understanding the information they found. This result is consistent with previous research that observed a positive relationship between individual level of income and health literacy [39–41]. This relationship was examined because lower income may negatively affect health literacy and thus increase the risk for poor health outcomes. Overall, the present study first identified a pro-rich concentration in health literacy score in China.

Consistent with previous studies, we found that health literacy was strongly associated with education [7]. Inadequate health literacy reflects a disparity in the multidimensions of healthcare and the healthcare system. For example, adults with limited health literacy obtained less health information and had less experience interacting with health professionals than the general population. Many studies lack a specific description of the nature of the disparity and potential explanatory pathways; thus, how health literacy contributes to disparities remains rather vague [14]. Our study further confirmed the assumption that compared with the education variable, health literacy can be a stronger correlate of individuals’ health status or even other socioeconomic factors.^2^

Regarding HISB from healthcare professionals, our results first showed a pro-rich distribution among the urban population. Compared with the high-income population, the low-income population was consistently less likely to use healthcare professionals as their first source for health information. In developed countries, studies assessing equity in health-information seeking have consistently identified personal characteristics such as income as the main determinant related to the HISB of patients. Our third result was that income inequalities contributed most to the pro-rich distribution of health-information seeking among the urban population, which was similar to the results of previous studies [42–44].

Health-information sources available to the public include communication with health professionals, communication with their family or friends, printed publications, and the internet [45]. Doctors and nurses were identified as the top information source for individuals who were categorised as seeking health information from online and offline sources. A growing number of studies have considered internet or online HISB [44, 46]. However, the network monopoly and the death of Wei Zexi due to misleading information from the internet challenged the online approach as a dominant source of health information in China [5]. In this case, a college student contacted a military hospital in Beijing through the search engine Baidu, which then provided cellular immunotherapy for his cancer. His family believed in the misguided advertisement that the treatment had ‘an efficacy rate of 80–90%’ and ‘could easily prolong my life for 20 years’, and thus spent all their savings. This event revealed the poor quality of health-information seeking from the internet for patients in China and highlights the importance of improving disparities in health-information access.

The Chinese government increased its spending on health from 11 to 29 dollars between 2000 and 2011. In China, four-fifths of government health spending is allocated to public healthcare providers to encourage universal health coverage. However, to ensure equitable health-information seeking and health literacy among patients for those in need is equally important for financial improvements. As we have presented in the introduction, primary health literacy is a one of key drivers in a successful interaction between healthcare providers and those who need healthcare from diagnosis to recovery. In a community where each one has access to easy-to-use health information, individuals can facilitate their health-related decisions and be able to take actions to prevent unhealthy behaviours and promote their health benefits. Therefore, it is vital for physicians and nurses to better communicate with all residents, not only patients, by accomplished increasing their health literacy [47]. Today, more and more online and community health activities make our world of information flat as well. Additional efforts to promote incorporation of health literacy in community and school education should call for partnership with internet workers, social workers and school staffs.

Our study has several limitations that can be improved on in further research. First, this is the first study to describe the general pattern and associated factors of disparity in health literacy and health-information seeking in China. More studies that analyse the process and shaping of health literacy among the population and detail health-information seeking between residents and healthcare professionals are warranted. Second, the data is from a survey that covers only the urban population. Thus, an analysis using a more representative data for urban and rural populations in China is required. Although our study revealed that urban residents perceived healthcare professionals as their main source of health information, further attention must be drawn to the rapid growth of online health-information seeking. Finally, we also consider that the “teach-back” techniques to check patients understanding of health information are equally important for health care services and providers. It would be a vital step for the next study.

## Conclusion

We conducted an analysis to examine the degree of income-related inequality in health literacy and health-information seeking and the contributions of the main socioeconomic determinants in China. Compared with the high-income population, the low-income population was consistently less likely to seek health information or to use healthcare professionals as its first source for health information and had more difficulty understanding the found information. Public interventions in China to reduce inequality in health literacy and HISBs among the urban population, coupled with easily accessible information sources on health, warrant further attention from policymakers.

## Abbreviations

BMI: Body mass index
CI: Concentration index
GCHS: Guangzhou Community Health Survey
HI: Horizontal index
HISB: Health-information seeking behaviour
HL: Health literacy
TB2: Type 2 diabetes

## Footnotes

1 We slightly modified the mix of questions because of two reasons: the one is that the questions about health literacy in our questionnaire is not a structured part targeted only health literacy, thus we could not find out exact questions corresponding to the published paper; the other is that nearly half of the women had no formal education in the surveyed sample in Wang et al [24] and that survey covered only rural women from a Chinese poor minority area, in contrast, our survey targeted only urban population in Guangzhou, one of top metro city in China.

2 We appreciate the reviewers’ comment on migrancy, ethnicity/language influencing health literacy and health seeking behaviour, unfortunately, the survey provided no such information.

## References

[1] Ishikawa H, Yano E (2008). Patient health literacy and participation in the health-care process. Health Expect.

[2] Anker AE, Reinhart AM, Feeley TH (2011). Health information seeking: a review of measures and methods. Patient Educ Couns.

[3] Jeong SH, Kim HK (2016). Health literacy and barriers to health information seeking: A nationwide survey in South Korea. Patient Educ Couns.

[4] Nutbeam D (2008). The evolving concept of health literacy. Soc Sci Med.

[5] Ouyang Y (2016). Student's death highlights gaps in China's health regulations. Lancet Oncol.

[6] Ad HCOH, American MA (1999). Health literacy: Report of the council on scientific affairs. JAMA.

[7] Berkman ND, Sheridan SL, Donahue KE, Halpern DJ, Crotty K (2011). Low health literacy and health outcomes: an updated systematic review. Ann Intern Med.

[8] Cho YI, Lee SD, Arozullah AM, Crittenden KS (2008). Effects of health literacy on health status and health service utilization amongst the elderly. Soc Sci Med.

[9] Schillinger D, Barton LR, Karter AJ, Wang F, Adler N (2006). Does literacy mediate the relationship between education and health outcomes? A study of a low-income population with diabetes. Public Health Rep.

[10] Doorslaer EV, Koolman X, Jones AM (2004). Explaining income-related inequalities in doctor utilisation in Europe. Health Econ.

[11] Pickett KE, Wilkinson RG (2015). Income inequality and health: A causal review. Soc Sci Med.

[12] Koh HK, Berwick DM, Clancy CM, Baur C, Brach C, Harris LM, et al. New federal policy initiatives to boost health literacy can help the nation move beyond the cycle of costly 'crisis care'. Health Affair. 2012;31:434–43. 10.1377/hlthaff.2011.1169 PMC5102007 22262723

[13] Finney Rutten LJ, Hesse BW, Moser RP, Ortiz Martinez AP, Kornfeld J, Vanderpool RC (2012). Socioeconomic and Geographic Disparities in Health Information Seeking and Internet Use in Puerto Rico. J Med Internet Res.

[14] Mantwill S, Monestel-Umaña S, Schulz PJ (2015). The Relationship between Health Literacy and Health Disparities: A Systematic Review. PLOS ONE.

[15] Wolf MS, Knight SJ, Lyons EA, Durazo-Arvizu R, Pickard SA, Arseven A (2006). Literacy, race, and PSA level among low-income men newly diagnosed with prostate cancer. Urology.

[16] Li X, Lu J, Hu S, Cheng KK, De Maeseneer J, Meng Q (2017). The primary health-care system in China. The Lancet.

[17] Adler NE, Newman K. Socioeconomic disparities in health: pathways and policies. Health Affair. 2017;21:60–76. 10.1377/hlthaff.21.2.60 11900187

[18] Paasche-Orlow MK, Wolf MS (2007). The causal pathways linking health literacy to health outcomes. Am J Health Behav.

[19] Lambert SD, Loiselle CG (2007). Health information—seeking behavior. Qualitative Health Res.

[20] Weaver JB, Mays D, Weaver SS, Hopkins GL, Eroğlu D, Bernhardt JM (2010). Health information–seeking behaviors, health indicators, and health risks. Am J Public Health.

[21] Miller SM (1989). Cognitive informational styles in the process of coping with threat and frustration. Adv Behav Res Ther.

[22] Pan B, Chen X, Wu X, Li J, Li J, Li Y (2014). Prevalence of Noncommunicable Diseases and Their Risk Factors in Guangzhou, China. Prev Chronic Dis.

[23] Wu X, Pan B, Chen X, Zhuang X, Zhu K, Zeng L (2014). Useful information for hypertension management reform in community health care: prevalence, awareness, treatment and control among Guangzhou adults. Clin Exp Hypertens.

[24] Lee SD, Tsai T, Tsai Y, Kuo KN (2010). Health literacy, health status, and healthcare utilization of Taiwanese adults: results from a national survey. BMC Public Health.

[25] Lam LT, Yang L (2014). Is low health literacy associated with overweight and obesity in adolescents: an epidemiology study in a 12–16 years old population, Nanning, China, 2012. Arch Public Health.

[26] Wang C, Li H, Li L, Xu D, Kane RL, Meng Q (2013). Health literacy and ethnic disparities in health-related quality of life among rural women: results from a Chinese poor minority area. Health Qual Life Outcomes.

[27] Xiao L, Ying-Hua LI, Chen GY, Yu MA, Jun-Feng HU, Cheng YL. Development of health literacy comprehensive index. Chin J Health Educ. 2009;25:103–05.

[28] Xiao L, Cheng YL, Ma Y, Chen GY, Hu JF, Li YH (2008). A study on applying Delphi method for screening evaluation indexes of health literacy of China adults. Chin J Health Educ.

[29] Wang C, Kane RL, Xu D, Meng Q (2015). Health literacy as a moderator of health-related quality of life responses to chronic disease among Chinese rural women. BMC Womens Health.

[30] Kim SH (2009). Health literacy and functional health status in Korean older adults. J Clin Nurs.

[31] O'Donnell O, O'Neill S, Ourti TV, Walsh B (2016). conindex: Estimation of concentration indices. Stata J.

[32] Wagstaff A, O'Donnell O, Van Doorslaer E, Lindelow M. Analyzing health equity using household survey data: a guide to techniques and their implementation. Washington, D.C.: The World Bank Publications, 2007.

[33] Deaton A, Zaidi S. Guidelines for constructing consumption aggregates for welfare analysis. Washington, D.C.: The World Bank Publications, 2002.

[34] Guangzhou Statistics Bureau. Guangzhou: Guangzhou Statistical Yearbook, 2014.

[35] Bakkeli NZ (2016). Income inequality and health in China: A panel data analysis. Soc Sci Med.

[36] Yang W, Kanavos P (2012). The less healthy urban population: income-related health inequality in China. BMC Public Health.

[37] Zhou Z, Su Y, Gao J, Campbell B, Zhu Z, Xu L (2013). Assessing equity of healthcare utilization in rural China: results from nationally representative surveys from 1993 to 2008. Int J Equity in Health.

[38] Elwell-Sutton TM, Jiang CQ, Zhang WS, Cheng KK, Lam TH, Leung GM (2013). Inequality and inequity in access to health care and treatment for chronic conditions in China: the Guangzhou Biobank Cohort Study. Health Policy Plann.

[39] Sudore RL, Yaffe K, Satterfield S, Harris TB, Mehta KM, Simonsick EM (2006). Limited literacy and mortality in the elderly: the health, aging, and body composition study. J Gen Intern Med.

[40] Baker DW, Gazmararian JA, Williams MV, Scott T, Parker RM, Green D (2002). Functional health literacy and the risk of hospital admission among Medicare managed care enrollees. Am J Public Health.

[41] von Wagner C, Knight K, Steptoe A, Wardle J (2007). Functional health literacy and health-promoting behaviour in a national sample of British adults. J Epidemiol Commun Health.

[42] Wang MP, Viswanath K, Lam TH, Wang X, Chan SS (2013). Social Determinants of Health Information Seeking among Chinese Adults in Hong Kong. PLOS ONE.

[43] Lalazaryan A, Zare-Farashbandi F (2014). A review of models and theories of health information seeking behavior. Int J Health Syst Disaster Manag.

[44] Nölke L, Mensing M, Krämer A, Hornberg C (2015). Sociodemographic and health-(care-) related characteristics of online health information seekers: a cross-sectional German study. BMC Public Health.

[45] Cotten SR, Gupta SS (2004). Characteristics of online and offline health information seekers and factors that discriminate between them. Soc Sci Med.

[46] Gutierrez N, Kindratt TB, Pagels P, Foster B, Gimpel NE (2014). Health literacy, health information seeking behaviors and internet use among patients attending a private and public clinic in the same geographic area. J Community Health.

[47] Powers BJ, Trinh JV, Bosworth HB (2010). Can this patient read and understand written health information?. JAMA.

